# Analysis of gene × environment interactions in sibships using mixed models

**DOI:** 10.1186/1471-2156-4-S1-S18

**Published:** 2003-12-31

**Authors:** Jill S Barnholtz-Sloan, Laila M Poisson, Steven W Coon, Gary A Chase, Benjamin A Rybicki

**Affiliations:** 1 Department of Internal Medicine (Oncology), Wayne State University School of Medicine and Karmanos Cancer Institute, 110 East Warren, Detroit, Michigan, USA; 2 Department of Biostatistics and Research Epidemiology, Henry Ford Health Sciences Center, One Ford Place, Detroit, Michigan, USA

## Abstract

**Background:**

Gene × environment models are widely used to assess genetic and environmental risks and their association with a phenotype of interest for many complex diseases. Mixed generalized linear models were used to assess gene × environment interactions with respect to systolic blood pressure on sibships adjusting for repeated measures and hierarchical nesting structures. A data set containing 410 sibships from the Framingham Heart Study offspring cohort (part of the Genetic Analysis Workshop 13 data) was used for all analyses. Three mixed gene × environment models, all adjusting for repeated measurement and varying levels of nesting, were compared for precision of estimates: 1) all sibships with adjustment for two levels of nesting (sibs within sibships and sibs within pedigrees), 2) all sibships with adjustment for one level of nesting (sibs within sibships), and 3) 100 data sets containing random draws of one sibship per extended pedigree adjusting for one level of nesting.

**Results:**

The main effects were: gender, baseline age, body mass index (BMI), hypertensive treatment, cigarettes per day, grams of alcohol per day, and marker GATA48G07A. The interaction fixed effects were: baseline age by gender, baseline age by cigarettes per day, baseline age by hypertensive treatment, baseline age by BMI, hypertensive treatment by BMI, and baseline age by marker GATA48G07A. The estimates for all three nesting techniques were not widely discrepant, but precision of estimates and determination of significant effects did change with the change in adjustment for nesting.

**Conclusion:**

Our results show the importance of the adjustment for all levels of hierarchical nesting of sibs in the presence of repeated measures.

## Background

Gene × environment models are widely used to assess genetic and environmental risks and their association with a phenotype of interest for many different complex diseases [1,2]. The Framingham Heart Study began in 1948 with the aim of gathering the longitudinal family data needed for a comprehensive study of genetic and environmental risks for cardiovascular disease. In 1971, a second-generation group was enrolled called the Offspring Cohort and has been followed every four years since. The Framingham data has led to the discovery of major cardiovascular risk factors (e.g., high blood pressure, high blood cholesterol, smoking, obesity, diabetes, physical inactivity), important related factors (e.g., age, gender, psychosocial factors, blood triglyceride, and lipid levels), as well as genetic risk factors [3].

Studies have examined environmental and genetic variables influencing blood pressure in the Framingham data as well as in other large epidemiologic data sets [3,4]. These studies were performed in extended pedigree, sibship, and case-control data. Genetic studies have found multiple regions on the genome that may contain a candidate gene for systolic blood pressure (SBP) and/or hypertension. Among these regions are areas on chromosomes 10 and 17 [5,6]. Chromosome 17 contains the angiotensin-I converting enzyme (ACE) gene and there is good supporting evidence that this gene is involved in hypertension [7-9].

Sibship studies are routinely used when parents and other members of the extended pedigree are not available for study. Full sibs share approximately half of their genome and usually share a common environment for a period of their lives, making them good candidates for gene × environment risk studies. Longitudinal studies, which involve taking measurements of the same factors over time on an individual, can be used to enhance the accurate assessment of gene × environment models in sib studies. With repeated measures data, stability over time of measurements can be evaluated, whereas in a cross-sectional study these measurements can only be evaluated at one point in time. However, repeated measures add complexity to variance estimation.

In addition to the correlation of data points collected in a serial manner, there is also the hierarchical nesting structure of sibs within sibships and of sibships within pedigrees. In cases when there is more than one sibship available per extended pedigree, most studies use either all sibships available, without accounting for the correlation between them, or one sibship only is randomly drawn from the extended pedigree and used for analysis. Mixed generalized linear models are frequently used to account not only for the situation in which there are repeated measures on an individual, but also when there is a complex hierarchical nesting structure present within the data. Hence, we used mixed generalized linear models to assess gene × environment interactions with respect to SBP on sibships from the Framingham Heart Study offspring cohort data available for the Genetic Analysis Workshop 13 (GAW13). We compared precision of estimates of significant effects obtained from using all available sibships, while controlling for the nesting of sibs within sibships and of sibships within extended pedigrees and repeated measures, to results from analyses in which the sibship nesting within extended pedigrees is either ignored or obviated by selection of one sibship per pedigree.

## Methods

### Study Population

Sibship data from the Framingham Heart Study offspring cohort (part of the GAW13 data) was used for all analyses, in which each sib could have participated in any or all of five exam periods. The data consisted of 410 sibships total (from 330 extended pedigrees) and for the data where one sibship was randomly drawn from each extended pedigree, 330 sibships were included. The phenotype of interest was SBP, in its continuous form. The covariates of interest were: baseline age, baseline gender and height, weight, hypertensive treatment, number of cigarettes per day, and number of grams of alcohol per day all measured over the five exam periods. Body mass index (BMI) was used rather than height and weight separately.

### Statistical Analysis

Mixed generalized linear models were implemented using PROC MIXED in SAS Version 8 software [10-12], to account for the repeated covariate measurements over the five exam periods and for the hierarchical nesting structure of sibs within sibships and of sibships within extended pedigrees. Markers of interest were chosen using FBAT [13,14], in which tests for association with high SBP for each multiallelic marker in the genome scan were performed using a dichotomous form of SBP (≥ 140 and < 140). Of the markers highly associated with high SBP (dominant and additive models, *p *= 0.05), three markers were used: GATA48G07A and GGAA5D10 on chromosome 10 and GGAA7D11 on chromosome 17, because previous studies showed association with locations on chromosomes 10 and 17 [5-9]. The SBP-associated alleles at the three markers were then pooled to result in 3, 4, and 3 alleles for markers GATA48G07A, GGAA5D10, and GGAA7D11, respectively, with all alleles not associated with SBP pooled into the last allele (see Table 1).

Univariable and multivariable models were fitted and decisions for inclusion/exclusion were based on statistical significance from the models. Explanatory factors shown to be significant in previous studies of SBP were also kept in the model [3,4], where the phenotype of interest was SBP in its continuous form. The results were used to develop a gene × environment model, using all sibship data available (410 sibships total from 330 extended pedigrees, where the average sibship has three individuals (median = 3)), adjusting for both nesting of sibs within sibships and nesting of sibships within extended pedigrees and adjusting for repeated measures. This gene × environment model was then fitted again to the same full data set, while adjusting for only one level of nesting (sibs within sibships) and for repeated measures. Then the gene × environment model was fitted to 100 data sets containing random draws of one sibship per extended pedigree (every pedigree is represented by one sibship; 60 out of 330 pedigrees has multiple sibships), adjusting for repeated measures and one level of nesting (sibs within sibships), in which estimate values, standard errors and *p*-values were averaged over all 100 data sets. The precision of parameter estimates for main and interaction effects were compared across all three nesting and repeated measures adjustment techniques by empirical evaluation of the resulting standard errors and *p*-values. For a specific effect, the estimate having the lowest standard error and *p*-value of the three models was determined to be the most precise estimate.

## Results

The main effects included in the model were: gender, baseline age, BMI, hypertensive treatment, cigarettes per day, grams of alcohol per day, and marker GATA48G07A (heterozygote genotype with alleles 1 (allele 350 bp) and 3 (all other alleles) (57% of total individuals) contrasted with all other possible genotypes). The interaction fixed effects were: baseline age by gender, baseline age by cigarettes per day, baseline age by hypertensive treatment, baseline age by BMI, hypertensive treatment by BMI, and baseline age by marker GATA48G07A (significant interactions from fitting all second-order interactions on the full data set). The estimates and standard errors for all main and interaction fixed effects included in the gene × environment model performed using all three nesting adjustment techniques with repeated measures adjustment are given in Table 2.

The model including all available sibships adjusting for both levels of nesting and repeated measures was the most precise. The standard errors of the estimates for the main and interaction fixed effects were smaller (or equal to) standard errors of the model fitted on the same data adjusting for one level of nesting only and the model fitted on 100 random draws of one sibship per pedigree, adjusting for one level of nesting. Across the 100 data sets with random draws of one sibship per extended pedigree, the effects and standard error estimates were highly stable (data not shown).

For most of the main and interaction fixed effects, as compared to the full data set adjusted for both levels of nesting and repeated measures, the *p*-values either increase (but are still statistically significant), or they increase such that the effect would no longer be considered statistically significant, when the model is fitted on the full data set adjusting for only one level of nesting or fitted on the data when one sibship per extended pedigree is drawn at random (see Table 2 with interest in cigarettes per day, BMI, marker GATA48G07A, baseline age and cigarettes per day, baseline age and BMI, hypertensive treatment and BMI, and baseline age and marker GATA48G07A).

## Discussion

Mixed generalized linear models allow for adjustment for repeated measures as well as hierarchical nesting in data. When performing an analysis on sib data from extended pedigrees within a longitudinal study, a modeling scheme needs to consider not only the correlation in repeated measurements on each individual, but also the correlation resulting from the nesting of sibs within sibships and the nesting sibships within the larger family structure. The precision and significance of model estimates vary depending on the adjustments that are used.

Using all data available, i.e., all possible sibships, we fitted a gene × environment model, adjusting for nesting of sibs within sibships and nesting of sibships within extended pedigrees and adjusting for repeated measures from each of five time periods. The model included all environmental main effects available, because they were either statistically or biologically essential to the prediction of SBP (see Table 2). The only univariable genetic effect that was significant was marker GATA48G07A (heterozygote genotype with alleles 1 and 3 as compared with all other possible genotypes) on chromosome 10, which is in a region that has previously been shown to be associated with SBP [5,6]. Multiple interaction effects were significant using the full data set with all adjustments (see Table 2). This model was then fitted to the full data set, adjusting for one level of nesting only and repeated measures and to 100 data sets consisting of random draws of one sibship per extended pedigree, adjusting for one level of nesting only and repeated measures.

The estimates for all three nesting techniques were not widely discrepant, but precision of estimates of significant effects did change with the change in adjustment for nesting, i.e., when all levels of nesting were adjusted for as well as repeated measures, the precision was at the highest level.

## Conclusions

Our results show the importance of the adjustments for all levels of hierarchical nesting of sibs in the presence of repeated measures in these analyses. We have shown that precision of estimates of statistically significant effects is negatively affected when all levels of a hierarchical nesting structure are not taken into account when using sibships from extended pedigree data.

## Figures and Tables

**Table 1:** Reassignment of allele names for the three markers of interest: GATA48G07A, GGAA5D10 and GGAA7D11

Marker	Allele No.	Alleles Pooled
GATA48G07A	1	350 bp
	2	362 bp
	3	All others
GGAA5D10	1	93 bp
	2	109 bp
	3	105, 121 bp
	4	All others
GGAA7D11	1	274 bp
	2	286 bp
	3	All others

**Table 2:** Adjusted fixed effects estimates and standard errors from the gene × environment model

	FULL DATA-ADJ2^A^			FULL DATA-ADJ1^B^			ONE SIBSHIP DATA-ADJ1 (100 random draws)^C^		
Effect	Estimate	SE	*P*-value (*t*-test)	Estimate	SE	*P*-value (*t*-test)	Avg Estimate	Avg SE	Avg *P*-value (*t*-test)

Intercept	95.72	6.91	<0.0001	95.99	6.90	<0.0001	87.67	7.81	<0.0001
Gender	-10.71	1.89	<0.0001	-9.98	2.07	<0.0001	-4.71	2.32	0.0001
Baseline age	0.15	0.14	0.29	0.19	0.14	0.18	0.16	0.16	0.36
BMI	0.49	0.16	0.003	0.51	0.16	0.002	0.63	0.18	0.001
Hypertensive treatment	-2.44	4.55	0.59	-3.32	4.60	0.47	-0.20	5.34	0.68
Cigarettes per day	0.11	0.05	0.04	0.13	0.05	0.02	0.10	0.06	0.11
Grams of alcohol per day	0.07	0.01	<0.0001	0.06	0.01	<0.0001	0.06	0.01	<0.0001
Marker GATA48G07A	5.49	2.24	0.01	4.04	2.07	0.05	2.84	2.33	0.02
Baseline age and gender	-0.19	0.06	0.0005	-0.17	0.06	0.006	-0.16	0.07	0.03
Baseline age and cigarettes per day	-0.005	0.002	0.003	-0.005	0.002	0.001	-0.004	0.002	0.01
Baseline age and hypertensive treatment	0.14	0.07	0.04	0.13	0.07	0.06	0.19	0.08	0.03
Baseline age and BMI	0.02	0.005	0.0005	0.02	0.005	0.001	0.02	0.006	0.008
Hypertensive treatment and BMI	-0.29	0.12	0.01	-0.30	0.12	0.01	-0.26	0.13	0.07
Baseline age and marker GATA48G07A	0.14	0.07	0.04	0.11	0.06	0.08	0.16	0.07	0.03

^A^FULL DATA-ADJ2: full data set using all available sibships adjusting for both levels of nesting (sibs within sibships and sibships within pedigrees); ^B^FULL DATA-ADJ1: full data set using all available sibships adjusting for one level of nesting (sibs within sibships); ^C^ONE SIBSHIP DATA-ADJ1 (100 random draws): data set using one randomly drawn sibship per each extended pedigree, adjusting for one level of nesting (sibs within sibships) performed 100 times.
